# Impact of Janus Kinase Inhibition on the Treatment of Axial Spondyloarthropathies

**DOI:** 10.3389/fimmu.2020.591176

**Published:** 2020-10-21

**Authors:** Ariane Hammitzsch, Georg Lorenz, Philipp Moog

**Affiliations:** Section of Rheumatology, Department of Nephrology, Klinikum Rechts der Isar, Technical University of Munich, Munich, Germany

**Keywords:** JAK – STAT signalling pathway, small molecule inhibitor, axial spondyloarthritis, preclinical efficacy and tolerability, safety profile

## Abstract

Many immune cells and effector molecules (e.g. cytokines, Interferons, growth factors) utilize different combinations of Janus kinase (JAK) and signal transducer and activator of transcription (STAT) molecules to transduce signals from the cell surface to the nucleus, where they regulate transcription. This pathway is basically involved in almost all inflammatory diseases and also in the interleukin (IL)-23/IL-17 cascade, which is an essential part of the pathogenesis of spondyloarthropathies (SpA). Upon evidence from *in vitro* and *in vivo* experiments indicating disease-modifying effects of JAK inhibition in inflammatory joint disease, numerous inhibitors of the JAK/STAT pathway (= JAKinibs) with different selectivity against the four members of the JAK family [JAK1, JAK2, JAK3, and tyrosine kinase 2 (TYK2)] were developed. Trials in rheumatoid arthritis were successful with respect to efficacy and safety, and currently, three JAKinibs are approved for the treatment of rheumatoid arthritis in the European Union. Although new treatment options (anti-IL-23, anti-IL-17, and phosphodiesterase 4 inhibitors) have become available for spondyloarthritis and especially psoriatic arthritis (PsA) within the last years, most of them are biologics and do not address all disease manifestations equally. Therefore, multiple trials were initiated to evaluate JAKinibs in PsA and axial spondyloarthritis (axSpA). A trial of Tofacitinib (OPAL) was successful in PsA and has led to the inclusion of JAKinibs into the treatment algorithm. Currently many trials with JAKinibs are ongoing for PsA and axSpA, with one phase III trial of upadacitinib (selective JAK1 inhibitor) showing good therapeutic response in active radiographic axSpA.

## Introduction

Spondyloarthropathies (SpA) are a group of chronic inflammatory diseases, including axial SpA (axSpA) and psoriatic arthritis (PsA), as well as other less common forms like enteropathic or reactive arthritis. Besides skeletal manifestations (axial disease, peripheral arthritis, enthesitis, and dactylitis), the involvement of extra-articular organs (uveitis, psoriasis, and inflammatory bowel disease [IBD]) is a shared feature of these diseases (1). Current therapeutic options for SpA are limited compared with those for rheumatoid arthritis (RA), especially for axSpA, and mainly antibody-based, such as anti-tumor necrosis factor (TNF), anti-Interleukin (IL)-23 and anti-IL-17. Additionally, the therapeutic response greatly varies between the different diseases and affected systems such as the spine, peripheral joints, skin, and eyes. Only 51.3% of axSpA patients respond to TNF inhibitors (TNFi), some loose response over time, and others are not eligible (2, 3). Janus kinase (JAK) and signal transducer and activator of transcription (STAT) molecules are central transmitters of pro- and anti-inflammatory signals in immune regulation (4). The IL-23/IL-17 pathway is highly important in the pathogenesis of SpA and is partly controlled by JAK (5, 6). Therefore, JAK inhibitors offer new treatment options for SpA. As these are currently more limited for axSpA compared with PsA, this review focuses on JAK inhibitors and their clinical application in axSpA.

## Janus Kinase and Signal Transducer and Activator of Transcription Signaling in Spondyloarthritis

**FIGURE 1:**
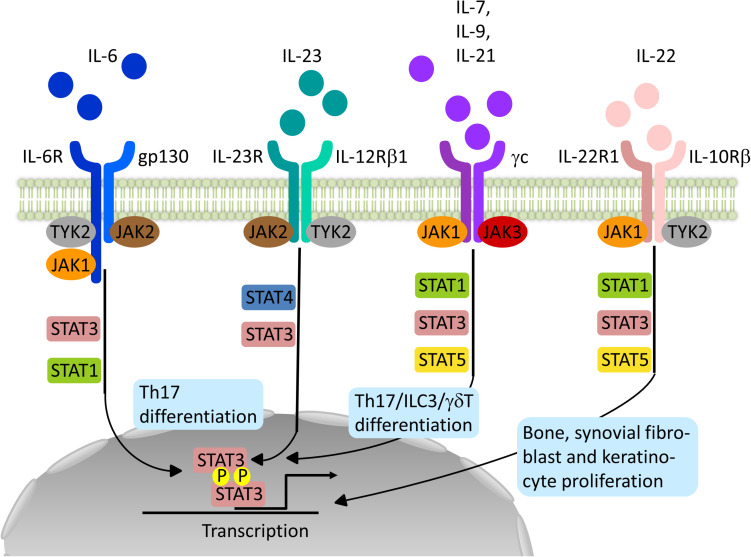
Schematic representation of relevant JAK-STAT signaling pathways in the pathogenesis of Spondyloarthritis. Binding of the different Interleukins (IL) to their specific receptor subunits on different cell populations, e.g. T cells, innate lymphoid cells (ILC) or effector cells such as osteoblasts, fibroblasts or keratinocytes leads to activation of a specific JAK-STAT pathway. The different isoforms of JAK are coupled to specific receptor/cytokine pairs and allow for a targeted inhibition with a specific JAKinib. However, overlap exists allowing for unintended side effects or accumulative effects. JAK1-specific inhibitors for example affect signaling by IL-6, IL-21, IL-7, IL-9 and IL-22 targeting most of the relevant immune and effector cell populations in SpA pathogenesis.

JAK and STAT are central signal transducers for a great number of pro-inflammatory (e.g. IL-2, IL-7, IL-12, and IL-23) and anti-inflammatory cytokines (e.g. IL-10) influencing innate immune responses thought to be essential for the induction of SpA and adaptive immune functions maintaining and perpetuating the disease (7–9). This intracellular tyrosine kinase family consists of JAK1, JAK2, JAK3, and tyrosine kinase 2 (TYK2) and is coupled to STAT molecules (STAT1, STAT2, STAT3, STAT4, STAT5a and b, and STAT6) (7). Cytokine-receptor binding on the cell surface leads to autophosphorylation of JAK or phosphorylation of a partner JAK. Such activated JAK further phosphorylate sites of the intracellular domain of the receptor providing docking sites for STAT molecules. Dimers of STAT molecules phosphorylated by JAK migrate to the nucleus where they regulate gene expression. Different combinations of JAK and STAT are assigned to different cytokines and their receptors, providing a multitude of pathways and functions, as depicted in Figure 1 (7). However, STAT can be activated by other kinases and exercise effects in an un-phosphorylated state and even extra-nuclear. JAK also act independently of STAT molecules, for example, by directly phosphorylating histones adding further to the complexity of JAK and STAT signaling in immune cell regulation (10).

With regard to the pathogenesis of SpA, JAKs are involved in the signaling of key cytokines within the IL-23/IL-17 pathway, and Genome-wide association studies have found single nucleotide polymorphisms (SNPs) for *IL23R*, *JAK2*, and *TYK2* in ankylosing spondylitis (AS) (11). IL-23, produced by activated myeloid cells, is important for the generation of IL-17 and IL-22 by target cells such as T helper cells 17 (Th17), gamma delta T cells (γδ T cells), or innate lymphoid cells (ILCs) type 3 (12). A combination of JAK2 and TYK2 transmits the IL-23 signal via STAT3 and, to a lesser extent, STAT4 (6, 13). IL-17A production is mainly JAK2-dependent, whereas IL-22 production requires TYK2 and JAK2 (14). By blocking IL-17 production, JAK inhibition subsequently affects the downstream effects of IL-17. Other cytokines favouring the development and maintenance of IL-17 producing cells include IL-6 (JAK1/JAK2/TYK2) and IL-21 (JAK1/JAK3) (7, 15). IL-22, another effector cytokine in the pathogenesis of SpA, uses the combination of JAK1 and TYK2 (7). Next to its protective functions at the epithelial barrier in the gut, IL-22 has pro-inflammatory and proliferative effects (synovial fibroblasts, keratinocytes) as well as osteoanabolic effects providing another interesting treatment target for SpA (16–18). Granulocyte-macrophage colony-stimulating factor, another pro-inflammatory cytokine produced by T cells and ILCs type 3 in SpA patients, signals via JAK2 (7, 19).

Respective of the various cytokines relying on JAK-STAT signaling, inhibition of this pathway offers multiple possibilities to modulate the immune and tissue response implicated in SpA. As cells of articular and extra-articular organs are utilizing this pathway, JAKinib will most likely affect the different sites of the disease. However, the protective pathways regulated by JAK-STAT might lead to adverse effects like viral infections by interfering with interferon signaling, as wells as with numbers and function of natural killer (NK) cells, cytotoxic T cells, and ILC (7).

## Animal and Preclinical Data on Janus Kinase Inhibitors in Spondyloarthropathies

Although animal models for SpA do not fully replicate human disease, they have been useful in examining the molecular disease mechanisms and the role of JAK-STAT signaling. Enthesitis, an early and common feature in all forms of SpA, appears in mice with a myeloid cell-specific A20 (TNF-α-induced protein 3) deficiency (9, 20). The SpA-like arthropathy in this model is independent of TNF and relies on IL-1β and IL-6. Treatment with tofacitinib, an unselective JAK inhibitor, significantly reduced disease activity, confirmed by less inflammation of the synovial–entheseal complex on histology (20). In the SKG mouse model, which resembles human SpA if arthritis is initiated with curdlan and is dependent on IL-23 and Th17 cells, treatment with tofacitinib ameliorated established disease (21). Another experimental JAKinib also suppressed both inflammation and periosteal/entheseal bone formation in this model (22).

JAKinibs of different selectivity were shown to reduce Th17-type responses in CD4 T cells from patients with AS, PsA, and RA *ex vivo* with similar efficacy (23). Small interfering RNA-mediated knockdown of TYK2, signaling downstream of IL-23, was shown to be equally efficient in reducing type-17 cytokine secretion compared with JAK1 silencing or tofacitinib treatment (23). Several SNPs around the *TYK2* locus are associated with AS. Some of these exonic SNPs lead to loss-of-function variants of TYK2. One of these SNPs associated with multiple autoimmune diseases is protective, but does not impact on non-autoimmune domains such as susceptibility to infections (24). A highly specific TYK2 inhibitor, NDI-031407, blocked disease progression in the SKG mouse model (14). MRI imaging showed prevention of joint space narrowing and bone marrow edema. NDI-031407 also protected mice from bone marrow edema and enthesis-related synovitis in the IL-23 mini-circle model (mostly dependent on γδ T cells). It completely abrogated IL-22 production but only partially inhibited IL-17 production from γδ T cells upon stimulation with IL-1β and IL-23 (14). The frequency of a loss-of-function *TYK2* SNP (rs12720356) was significantly higher in AS patients with lower rates of spinal fusion, providing further evidence that targeting JAK could have effects on ankylosis.

With regard to the effects of JAKinib on bone metabolism, an important aspect in SpA, an experimental JAK2 inhibitor, AG490, reduced alkaline phosphatase activity in primary bone-derived cells from AS patients and healthy controls (25). On the other hand, tofacitinib and baricitinib increased bone mass in the K/BxN serum-transfer mouse model of RA-like arthritis and enhanced osteoblast function *in vitro* while sparing osteoclasts (26). These findings were confirmed in two RA patients treated with tofacitinib showing a substantial reduction in erosions of the metacarpophalangeal joints by micro-CT. Because activation of bone formation is deleterious in axSpA but useful in RA, further insight into the differential effects of JAKinib in these diseases is warranted.

Considering the combined data from animal models and clinical trials of anti-IL-23 antibodies in axSpA, TYK2 and JAK1 emerge as most promising targets of JAKinib for the treatment of axSpA, as they are involved in pathways relevant to the initiation (IL-23) and effector (IL-22) phase of the disease and especially in osteoproliferation (27). Table 1 gives an overview of JAKinibs already tested in clinical trials and under preclinical evaluation.

**TABLE 1:** Overview of JAK inhibitors tested in clinical trials and under preclinical evaluation for spondyloarthropathies and related diseases.

**JAK inhibitor**	**Target**	**Trial/disease**	**Status**	**PMID/NCT trial number**	**Trial status**
Tofacitinib	JAK1/JAK3	Rheumatoid Arthritis	Approved	(28–31)	
		Psoriatic Arthritis	Approved	(32, 33)	
		Axial Spondyloarthritis	Phase III	NCT03502616	Active
		Ulcerative Colitis	Phase III	(34)	
		Crohn’s disease	Phase II	(35)	
		Psoriasis	Phase III	(36)	
		Uveitis	Phase II	NCT03580343	Active
Baricitinib	JAK1/JAK2	Rheumatoid Arthritis	Approved	(37, 38)	
Filgotinib	JAK1	Rheumatoid Arthritis	Phase III	(39)	
		Axial Spondyloarthritis	Phase III	NCT04483687, NCT04483700	Not started, Not started
		Psoriatic Arthritis	Phase III	NCT04115748, NCT04115839	Active, Recruiting
		Ulcerative Colitis	Phase III	NCT02914522	Completed
		Crohn’s disease	Phase III	NCT02914561	Recruiting
		Uveitis	Phase II	NCT03207815	Recruiting
Upadacitinib	JAK1	Rheumatoid Arthritis	Approved	(40–42)	
		Axial Spondyloarthritis	Phase III	NCT04169373	Recruiting
		Psoriatic Arthritis	Phase III	NCT03104374, NCT03104400	Active, Active
		Ulcerative Colitis	Phase III	NCT03653026, NCT02819635	Recruiting, Recruiting
		Crohn’s disease	Phase III	NCT03345836, NCT03345849	Recruiting, Recruiting
Pefacitinib	Pan-JAK	Rheumatoid Arthritis	Phase III	(43, 44)	
		Psoriasis	Phase II	(45)	
		Ulcerative Colitis	Phase II	(46)	
Deucravacitinib (BMS-986165)	TYK2	Psoriatic Arthritis	Phase II	NCT03881059	Active
		Psoriasis	Phase II	(47)	
Abrocitinib (PF-04965842)	JAK1	Psoriasis	Phase II	(48), NCT02201524	Terminated
Itacitinib (INCB039110)	JAK1	Rheumatoid Arthritis	Phase II	NCT01626573	Completed
		Psoriasis	Phase II	NCT01634087	Completed
PF-06651600	JAK3	Rheumatoid Arthritis	Phase II	NCT04413617	Not started
		Ulcerative Colitis	Phase II	NCT02958865	Recruiting
		Crohn’s disease	Phase II	NCT03395184	Recruiting
SHR0302	JAK1	Rheumatoid Arthritis	Phase III	NCT04333771	Not started
		Axial Spondyloarthritis	Phase II/III	NCT04481139	Not started
		Ulcerative Colitis	Phase II	NCT03675477	Recruiting
		Crohn’s disease	Phase II	NCT03677648	Recruiting
PF-06826647	TYK2	Psoriasis	Phase II	NCT03895372	Recruiting
		Ulcerative Colitis	Phase II	NCT04209556	Recruiting
Brepocitinib (PF-06700841)	JAK1/TYK2	Psoriatic Arthritis	Phase II	NCT03963401	Active
		Psoriasis	Phase II	NCT02969018	Completed
		Ulcerative Colitis	Phase II	NCT02958865	Recruiting
		Crohn’s disease	Phase II	NCT03395184	Recruiting
NDI-031407	TYK2	SKG mouse model	Preclinical	(14)	
NDI-031232	TYK2		Preclinical		
SAR-20347	JAK1/TYK2	Psoriasis mouse model	Preclinical	(49)	

## Clinical Data on Janus Kinase Inhibitors in Spondyloarthropathies

Tofacitinib (a pan-JAKinib, 196 biologic naïve patients) and filgotinib (a selective inhibitor of JAK1, no more than one TNF inhibitor, 107 patients, TORTUGA) have been trialed in phase II trials of active AS with an inadequate response to ≥2 or intolerance to non-steroidal anti-inflammatory drugs and high-sensitivity C-reactive protein (CRP) ≥3 mg/L (filgotinib trial) (50, 51). Upadacitinib (selective for JAK1) has been evaluated in a combined phase II/III trial (178 JAKinib and biologic naïve patients, SELECT-Axis 1) in active AS with the earlier mentioned inclusion criteria (52). The combined data on the efficacy on disease activity, functionality, and radiographic progression summarized below are extracted from these studies.

### Efficacy on Disease Activity

After 12 weeks of treatment, Assessment in SpondyloArthritis International Society 20 (ASAS20) response rates were significantly higher for 5-mg tofacitinib twice daily (80.8%) and 200-mg filgotinib once daily (76%) compared with placebo (41.2 and 40%, respectively) but not for 2 mg (51.9%) or 10 mg (55.8%) of tofacitinib. ASAS40 response was significantly higher for all tofacitinib groups at week 12 and for 15-mg upadacitinib once daily compared with placebo at week 14 (52 vs. 26%). Tofacitinib (5-mg), filgotinib, and upadacitinib additionally lead to a significantly higher change of the mean Ankylosing Spondylitis Disease Activity Score (ASDAS) with rates of −1.4, −1.47 at week 12, and −1.45 at week 14, respectively, compared with placebo (−0.9, −0.57, and −0.54). Bath Ankylosing Spondylitis Disease Activity Index 50 (BASDAI50) response rates were significantly higher for all tofacitinib groups and upadacitinib with 42.3 to 46.2 and 45% vs. 23.5 and 23% in the placebo group. Enthesitis was significantly ameliorated by week 12 in 5- and 10-mg tofacitinib versus placebo.

The onset of response was slower with tofacitinib (approx. week 4) compared with TNF inhibitors but very rapid for filgotinib (week 1) and upadacitinib (week 2).

One limitation of the study of filgotinib is the relatively high proportion of patients with high high-sensitivity CRP at baseline, as elevated CRP is a known predictor of good response to therapy (53).

### Efficacy With Regard to Functionality

Spinal mobility measured by Bath Ankylosing Spondylitis Metrology Index (BASMI) improved significantly with filgotinib compared with placebo by week 12 (−0.75 vs. −0.39). In the tofacitinib trial, significant improvement of BASMI was only achieved with 10 mg twice daily. In the upadacitinib trial, consistent improvements were seen with treatment for BASMI but did not meet significance based on multiplicity adjustment per the Hochberg procedure.

### Efficacy With Regard to Radiographic Progression

Five- and 10-mg tofacitinib and filgotinib significantly improved Spondyloarthritis Research Consortium of Canada spine (−5.5 and −6.6, and −5.7) and sacroiliac joint (SIJ) scores (−3.2 and −3.6, and −3.52) compared with placebo (spine −0.1 and −0.52, SIJ −0.8 and 0.06). Upadacitinib had significant effects on the Spondyloarthritis Research Consortium of Canada spine score (−6.93 vs. −0.22).

A recent evaluation of the baseline and week 12 MRI scans from the TORTUGA trial found decreased SIJ erosion scores and increased backfill scores in the filgotinib group with increased erosion scores and no change in backfill scores in the placebo group, supporting the effects of filgotinib on structural lesions in axSpA (54).

### Treatment-Emergent Adverse Events

Treatment-emergent adverse events (TEAEs) appeared slightly more often with 5- and 10-mg tofacitinib compared with 2 mg and placebo (53.8 and 51.9% vs. 44.2 and 43.1%) and upadacitinib (62 vs. 55% in the placebo group) but were similar in the filgotinib trial (31% both groups). The most common TEAEs in all trials were nasopharyngitis and upper respiratory tract infections. There were no malignancies, opportunistic infections, and cases of active tuberculosis or cases of extra-articular manifestations (IBD, psoriasis, and uveitis). Episodes of herpes zoster (HZ) were reported with tofacitinib and upadacitinib, and one non-serious venous thromboembolic event (VTE) with filgotinib.

Results from a phase III randomized controlled trial (RCT) of tofacitinib in active AS (NCT03502616) are expected this year.

### Safety of Janus Kinase Inhibitors

In general, the long-term safety profile of JAKinibs is good and similar among the different inhibitors. Fears of high rates of opportunistic (including tuberculosis) and other infections have not been confirmed. Due to a lack of long-term data for JAKinib in SpA, data reported here are collected from clinical trials and post-marketing surveillance of RA. This seems feasible, as the three performed trials of JAKinibs in AS have so far shown similar safety profiles. However, it cannot be excluded that with longer observation periods and new trials leading to more exposed patients, new safety concerns may arise.

Overall incidence rates of serious infections are similar to those with biological disease-modifying antirheumatic drugs and range from 2.5 to 3.8 per 100 patient-years (55–58). However, a thorough screening of patients for tuberculosis before therapy is mandatory, with special alertness to extrapulmonary manifestations of tuberculosis (59). Reactivation of hepatitis B has been reported with JAKinib treatment, but treatment with tofacitinib in refractory cases under antiviral prophylaxis seems safe and effective (60, 61). The increased incidence of HZ is specific for JAKinib treatment and, for unknown reasons, seems to be more pronounced in Japan and Korea, ranging from 3.3 to 3.9 per 100 patient-years (26, 57, 58, 62, 63). The common risk factor for HZ over the different JAKinib was age (64, 65). Filgotinib so far has shown lower incidence rates of HZ and serious infections compared with other JAKinib, but long-term observations are lacking (66). This effect can possibly be attributed to less inhibition of JAK1-mediated signaling of interferon γ and IL-2, IL-4, and IL-15 (necessary for proliferation of NK cells) by filgotinib (67).

Regardless of a slightly increased risk for overall malignancies for RA patients compared with the general population, so far, no significant effects of JAKinib have been identified, excluding non-melanoma skin cancer (68). With respect to the two- to threefold increased risk for lymphoma in RA patients, the crude incidence rates with tofacitinib and baricitinib were low, with 0.10 (56). The effects of long-term use of JAKinib on the risk of cancer, for example, via interference with tumor surveillance through NK cells and interferon signaling are still unknown (57, 69).

As patients with RA, AS, and PsA generally have an increased risk for deep vein thrombosis (DVT), pulmonary embolism (PE), and venous thrombembolism (VTE) (risk ratios, 2.08, 2.17, and 1.96, respectively), special interest was given to such events in trials with JAKinibs (70–72). Incidence rates for DVT and PE with tofacitinib were 0.1 each (0.2 for PE with the 10-mg dose) and for VTE with upadacitinib 0.6 per 100 patient-years, and 0.1 and 0.2 per 100 patient-years with 100 and 200 mg of filgotinib (57, 58, 73). Therefore, a randomized safety endpoint study in moderate to severe RA comparing tofacitinib and TNF inhibitor has been implemented, including patients with at least one cardiovascular risk factor (NCT02092467). Nevertheless, the FDA and EMEA requested a warning of thrombosis for tofacitinib, baricitinib, and upadacitinib in 2019 (74, 75). A mechanistic explanation for the increased risk of thromboembolic events is still lacking. Despite a metanalysis of 30 RCTs on JAKinib in RA showing no significant differences in short-term major adverse cardiac events or VTE, a recent analysis of the World Health Organization global database (VigiBase) revealed a 2.3–3.4-fold increased risk for DVT and PE with tofacitinib and baricitinib in Europe (76, 77).

Use of a JAKinib has to be carefully evaluated in patients with risk factors for gastrointestinal perforation such as older age, history of diverticulitis or other gastrointestinal conditions, and use of prednisolone >7.5 mg/day or non-steroidal anti-inflammatory drugs (78, 79). The incidence rates per 1,000 patient-years for gastrointestinal perforation were 0.11 for tofacitinib and 0.04 for baricitinib (56). In analogy to tocilizumab, the risk of gastrointestinal perforation might be ascribed to the inhibition of IL-6 signaling by the different JAKinibs (57, 80).

Dose adjustments according to the metabolism of each drug should be considered for patients with moderate to severe hepatic or renal dysfunction. Laboratory changes in patients treated with JAKinibs are common and include changes of hemoglobin, lymphocyte and platelet counts. However, it has been hard to separate the intrinsic effects of JAKinibs via concomitant JAK2 inhibition (main signaling JAK for erythropoietin and thrombopoietin receptors) and disease-driven inflammatory effects on erythro- and thrombopoiesis. Other common laboratory changes include elevation of serum transaminases, creatinine, high- and low-density lipoprotein cholesterols, but usually do not result in treatment cessation.

Teratogenic effects of JAKinib have been reported in preclinical animal studies, and so far pregnancy outcomes of 47 patients treated with tofacitinib during RCT are known (81–83). There were 25 healthy newborns, one congenital pulmonary valve stenosis, seven spontaneous abortions, eight medical terminations, and six pending or lost to follow-up (84). Therefore, JAKinibs are contraindicated during pregnancy and breastfeeding, requiring strict contraception in females of child-bearing age until at least 1 week after the last dose.

Next to the known TEAEs of conventional synthetic and biological disease-modifying antirheumatic drugs, e.g. infections, a special focus has to be placed on HZ, VTE and PE, and changes in blood cells and lipid metabolism with JAKinib treatment.

## Discussion and Perspective

Although the data from three RCTs of JAK inhibitors in active AS are very promising, studies evaluating patients who have failed TNFi or anti-IL-17 therapy will be of great interest to place JAKinibs in the treatment algorithm of axSpA. Other interesting issues are head-to-head comparisons with TNFi and efficacy in non-radiographic axSpA. For a better assessment of the long-term safety results of the SpA study extensions will have to be awaited. Also the differential effect of more selective JAKinibs on the various disease manifestations of SpA needs to be clarified. Interest focuses on the newly developed specific TYK2 inhibitor, BMS-986165, which has already completed a phase II trial for psoriasis and promises clinical efficacy in axSpA by preclinical data and translational research. It also needs to be elucidated if SpA patients might profit from different dosing strategies for induction and maintenance of remission, such as high loading doses and low maintenance doses. However, these new orally available agents will most likely soon be included in the treatment recommendations for axSpA and provide the clinician with options in patients who are not eligible or have contraindications to TNFi or anti-IL-17, such as allergic reactions, congestive heart failure, or concomitant demyelinating disease (TNFi) and concomitant active IBD (anti-IL-17) (85, 86). JAKinibs may also be advantageous in patients with repeated infections, as they have a shorter half-life compared with bDMARD or csDMARD. With regard to avoiding radiographic progression and chronic disability in axSpA patients, JAKinibs face the same challenges as other drugs. From long-term observations with TNFi, it became evident that a halt in radiographic progression probably can only be achieved with very early and prolonged treatment (for more than 4 years) (87, 88). Targeting new bone formation specifically might have too many adverse effects on general bone homeostasis.

Overall, JAKinibs seem safe when used in a well-screened patient population of SpA and under regular surveillance. They appear equally effective to biologic drugs by current evidence and have advantages besides their oral application and shorter half-life.

## Author Contributions

AH and PM drafted the manuscript. AH wrote the manuscript and created graphical illustrations with the input from GL and PM. All authors approved the final version of the manuscript.

## Conflict of Interest

The authors declare that the research was conducted in the absence of any commercial or financial relationships that could be construed as a potential conflict of interest.
